# Evaluation of the levels of Fractalkine (CX3CL1), TNF-α, and TGF-β in the gingival crevicular fluid/tissue of patients with gingival overgrowth: a cross-sectional observational study

**DOI:** 10.1590/1678-7757-2025-0304

**Published:** 2025-11-27

**Authors:** Nazife HAMURCU, Ahu Uraz ÇÖREKCI, Rahşan Ilikçi SAĞKAN, Serpil CULA, Deniz Özbay ÇETINER

**Affiliations:** 1 Gazi University Faculty of Dentistry Department of Periodontology Ankara Turkey Gazi University Faculty of Dentistry, Department of Periodontology, Ankara, Turkey.; 2 Department of Periodontology Faculty of Dentistry Izmir Democracy University Izmir Turkey Department of Periodontology, Faculty of Dentistry, Izmir Democracy University, Izmir, Turkey.; 3 Uşak University Faculty of Medicine Department of Basic Medical Sciences Uşak Turkey Uşak University Faculty of Medicine, Department of Basic Medical Sciences, Medical Biology, Uşak, Turkey.; 4 Baskent University Faculty of Commercial Sciences Department of Insurance and Risk Management Ankara Turkey Baskent University, Faculty of Commercial Sciences, Department of Insurance and Risk Management, Ankara, Turkey.

**Keywords:** CX3CL1, Gingival overgrowth, Amlodipine, Cytokine

## Abstract

Fractalkine (CX3CL1) is expressed by various cells, contributing to the pathogenesis of diseases such as diabetes mellitus, vascular pathologies, and rheumatoid arthritis via immunological mechanisms. The CX3CL1–CX3CR1 axis regulates cellular responses such as proliferation and collagen production, which are implicated in gingival overgrowth (GO).

**Objectives:**

This study aimed to assess the levels of CX3CL1, tumor necrosis factor-α (TNF-α), and transforming growth factor-beta (TGF-β) in both gingival crevicular fluid (GCF) and gingival tissues among patients with biofilm- and amlodipine-induced GO. Additionally, the potential relationship between these biomarkers and clinical periodontal parameters was evaluated.

**Methods:**

The study included 17 participants with biofilm-induced GO (Group I), 18 participants with amlodipine-induced GO (Group A), and 10 systemically healthy participants without GO (Control). CX3CL1, TNF-α, and TGF-β levels in GCF samples were assessed using enzyme-linked immunosorbent assay (ELISA). Moreover, mRNA expression levels of CX3CL1, TNF-α, and TGF-β in tissue samples were determined by quantitative real-time PCR (qPCR).

**Results:**

The total GCF CX3CL1 level was significantly higher in Group I and Group A compared to controls. However, tissue CX3CL1 and TNF-α levels were significantly higher in Group I than Group A (p<0.05). In Group A, total GCF CX3CL1 levels showed a positive correlation with the gingival index (GI) (r=0.644), bleeding on probing (BOP) (r=0.622), and GCF volume (r=0.720). A significant positive correlation was observed between tissue CX3CL1 and TNF-α levels (r=0.762) (p<0.05). In Group I, a significant correlation was observed between total GCF CX3CL1 and TNF-α, TGF-β, and GCF volume levels, respectively (r=0.865, r=0.845, r=0.651). A positive correlation (p<0.05) was also found between tissue CX3CL1 and TNF-α and TGF-β levels, respectively (r=0.689, r=0.903).

**Conclusion:**

CX3CL1 may have a potential role in the development of GO-associated tissue fibrosis and its inflammatory mechanisms.

## Graphical abstract

**Figure:**
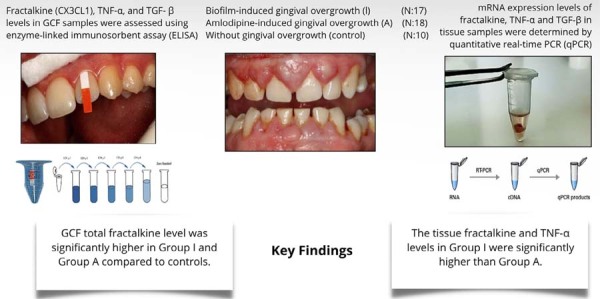

## Introduction

The gingival response to oral microbial biofilm can be influenced by systemic conditions and medications. This response may manifest as an increase in tissue density and/or gingival overgrowth (GO).^1^ Most GO cases result from adverse effects of systemic medications.^2^ Amlodipine, a third-generation dihydropyridine calcium antagonist, is known to cause drug-induced GO.^3^ Although the primary mechanism underlying amlodipine-induced GO has not been completely clarified, it is defined as a multifactorial process involving both inflammatory and non-inflammatory mechanisms.^4^

Fractalkine (CX3CL1) is the sole member of the CX3C sub-family.^5^ CX3CL1 exists in both soluble and membrane-bound forms and, unlike other chemokines, exhibits both chemotactic and adhesive properties. Soluble CX3CL1 is produced by the limited proteolysis of disintegrins, metallopeptidase 10 (ADAM10), and tumor necrosis factor-α-convertase enzyme (ADAM17/TACE), acting as a chemoattractant for monocytes, natural killer (NK) cells, and T cells. The membrane-bound form mediates integrin-independent leukocyte adhesion.^6^ CX3CL1 expression can be induced by various inflammatory cytokines, including transforming growth factor-beta (TGF-β), tumor necrosis factor-α (TNF-α), *Interleukin-1 beta* (IL-1β), and interferon-γ (IFN-γ), as well as by bacterial virulence factors such as lipopolysaccharide (LPS) from *P. gingivalis* and by alterations in oxygen levels.^5,7^ CX3CL1 is primarily produced by endothelial cells, neurons, synoviocytes, and monocytes, but it is also synthesized by keratinocytes, osteoblasts, and gingival fibroblasts.^8^ CX3CR1, a G-protein coupled receptor, is the only known receptor for CX3CL1 and is most prominently expressed by monocytes, macrophages, T cells, NK cells, osteoclasts, and gingival fibroblasts.^9^ The presence of CX3CL1 and CX3CR1 has been demonstrated in various systemic inflammatory diseases, in which they are essential for inflammatory cell migration, adhesion, and proliferation.^5^

Studies report that TNF-α, a pleiotropic cytokine with strong pro-inflammatory effects, significantly increases CX3CL1 expression. CX3CL1 mRNA levels in human umbilical vein endothelial cells (HUVECs) were shown to rise significantly after TNF-α stimulation.^7^ TGF-β is a crucial cytokine involved in fibrosis and has also been extensively evaluated for all types of GO, as it exhibits both pro-inflammatory and anti-inflammatory properties.^10^ Studies suggest the involvement of the CX3CL1–CX3CR1 axis in various fibrotic disorders. Animal model investigations reveal that this axis directly influences collagen production in processes such as systemic sclerosis, obstructive nephropathy, and liver fibrosis.^11-13^ Additionally, in primary cultures of rat microglia, stimulation with TGF-β—a critical mediator in idiopathic pulmonary fibrosis (IPF)—has been shown to elevate CX3CR1 mRNA levels, indicating a potential increase in CX3CR1 within TGF-β-enriched microenvironments.^14^ While the association of CX3CL1 with various diseases has been demonstrated, information regarding its role in periodontal diseases remains limited. Recent studies have highlighted its association with periodontitis by assessing its expression in various biological samples—including GCF, saliva, gingival tissue, and serum—as a possible inflammatory biomarker.^15-18^ Increasing evidence indicates that the CX3CL1–CX3CR1 signaling pathway is closely associated with the pathological process of fibrosis in multiple tissues and organs. The primary pathological hallmark of fibrosis, a dynamic process, is the abnormal synthesis and deposition of extracellular matrix (ECM).^19^ The CX3CL1–CX3CR1 axis contributes to the initiation and progression of fibrosis by means of upstream molecules such as TNF-α, TGF-β, and downstream molecules such as mitogen-activated protein kinase (MAPK), nuclear factor kappa B (NF-κB), phosphatidylinositol-3-kinase/protein kinase B (PI3K/AKT), and extracellular-regulated kinase (ERK). These pathways interact with epithelial cells and macrophages, thereby altering ECM deposition.^12^ Considering these processes, our study aimed to reveal the role of CX3CL1 in the pathogenesis of GO and its relationship with TNF-α and TGF-β, which are known to participate in inflammatory and fibrotic processes. To our knowledge, no previous study has explored the association between CX3CL1 with GO. Hence, this study sought to ascertain CX3CL1 levels in GCF and gingival tissues in individuals with biofilm- and amlodipine-induced GO. Furthermore, our investigation seeks to elucidate the interplay among TNF-α, TGF-β, and CX3CL1, as well as their correlation with clinical periodontal parameters.

## Methodology

### Study population

The study was approved by the Ethics Committee of Gazi University Faculty of Dentistry (GÜDHKAEK.19.08/2; April 18, 2019) and was conducted in compliance with the principles of the 1975 Declaration of Helsinki, as revised in 2013. Before participation, the purpose and content of the study were explained to all patients, who then signed an informed consent form confirming their voluntary participation. Demographic information (i.e., age and gender) was collected and recorded prior to clinical examination. This cross-sectional observational study was conducted at the Department of Periodontology, Faculty of Dentistry, Gazi University, and included 45 participants aged 18–60 years who were recruited from December 2018 to July 2019.

### Inclusion and exclusion criteria

Participants aged ≥ 18 years, non-smokers, with no radiographic bone loss around their existing teeth, and with GO in the maxillary/mandibular anterior region were included in the study. Individuals with plaque-induced GO were included in the biofilm group, whereas patients with GO who used a calcium channel blocker containing amlodipine 5 mg daily for at least six months constituted the amlodipine-induced GO group. Individuals with no GO and diagnosed with esthetic crown lengthening were selected as the control group. Exclusion criteria included a history of periodontal treatment within the previous six months, use of local or systemic antibiotics or anti-inflammatory drugs that could affect the inflammatory response, pregnancy or lactation, presence of caries, restorative treatment, or orthodontic appliances in the affected areas.

### Clinical examination

Participants were categorized into three groups: 17 individuals with biofilm-induced (inflammatory) GO (Group I), 18 individuals with amlodipine-induced GO (Group A), and 10 systemically healthy individuals without GO (Control).^20^ During the initial examination, full-mouth clinical measurements were conducted on all teeth, except for the third molars, using a Williams periodontal probe (Nordent Manufacturing Inc, Elk Grove Village, IL, USA). All measurements were made by a single calibrated examiner (NH). Calibration was assessed by measuring the probing pocket depths of 20 patients not included in the study groups, twice within 48 hours. Cohen’s kappa statistics was used to determine the reproducibility of the measurements, yielding an agreement value of 0.80. The recorded measurements included the plaque index (PI),^21^ gingival index (GI),^22^ probing depth (PD), bleeding on probing (BOP),^23^ clinical attachment level (CAL), and gingival overgrowth index (GOI)^24^. The degree of GO was assessed using the index described by Miller and Damm (MD).^24^ The height of the gingival tissue was vertically measured from the cemento-enamel junction (CEJ) to the free gingival margin. The following grades were recorded at six points around each tooth : 0 = normal gingiva; 1 = minimal GO (<2 mm), extending to the cervical third of the crown; 2 = moderate GO (2–4 mm), reaching the middle third of the crown; 3 = severe GO (>4 mm), extending to two-thirds of the crown. The mean GOI for each patient was determined by dividing the total GOI score of the measured surfaces by the number of teeth measured. Participants with second- and third-degree GO according to the GOI in the maxillary/mandibular anterior region were included. GCF and tissue samples were collected from patients one week after clinical measurements.^25^

### GCF sampling

GCF samples were preferably collected during the morning. Based on clinical measurements of the 35 patients in the biofilm and amlodipine group, regions with second (moderate) and third (severe) degree GO according to the GOI in the maxillary/mandibular anterior region were selected for GCF sampling. In the control group, sampling areas corresponded to sites where aesthetic crown lengthening would be performed in 10 patients. Before sample collection, the selected areas were isolated from saliva using sterile cotton rolls to prevent contamination. The supragingival biofilm was removed and dried with air spray without causing mechanical trauma to the tooth. GCF samples were obtained using specially prepared 2×8 mm paper strips (Periopaper^®^ Oraflow Inc. Smithtown, New York, USA), with four paper strips for each site. Each strip was inserted into the gingival crevice for 30 seconds until moderate pressure was felt. Strips contaminated with saliva and blood were excluded. Sample volumes were calculated and converted to µl.^26^ The tube lids were sealed with parafilm to prevent environmental contamination, and all samples were stored at -30°C until analysis.

### Gingival tissue sampling

In the GO patient groups, tissue samples were obtained from the same sites used for GCF collection. Under local anaesthesia, 1–2 mm subgingival incisions were made with a number 15 scalpel blade to include the gingival margin, sulcular epithelium, and connective tissue.^15^ In the control group, gingival tissues were collected during the aesthetic crown lengthening procedure following GCF collection. All samples were placed in polypropylene sample tubes containing 0.5 ml of RNA stabilization solution (RiboSave, GeneAll, Seoul, Korea) and stored at -80°C until analysis.

### Biochemical evaluation

CX3CL1, TNF-α, and TGF-β levels in GCF were analyzed using commercially available enzyme-linked immunosorbent assay (ELISA) kits, following the manufacturer’s instructions and employing the sandwich ELISA method (Bt-Laboratory Human CX3C- chemokine/ CX3CL1 ELISA Kit, sensitivity level 0.051 ng/ml, Human Tumour Necrosis Factor Alpha ELISA Kit, sensitivity level 1.52 ng/ml, Human Transforming Growth Factor Beta 1 ELISA Kit, sensitivity level 5.11 ng/ml, Shanghai, China). Optical density readings were performed using an ELISA plate reader (Thermo Fisher Scientific Multiskan, Massachusetts, USA). GCF cytokine concentrations were expressed in ng/ml, and the total amount of each cytokine was calculated as pg units by multiplying the concentration by the GCF volume.

### RNA isolation and cDNA synthesis of gingival tissue samples

Total RNA was isolated from gingival soft tissue samples using a total RNA purification kit (GeneAll^®^ Hybrid-R [305-101], Geneall Biotechnology Co., Seoul, Korea) according to the manufacturer’s recommendations. Complementary DNA (cDNA) synthesis was reverse transcribed using random Primers (GeneAll HyperScriptTM, Seoul, Korea) with the High Capacity cDNA Reverse Transcription Kit (Applied Biosystems, Foster City, CA, USA).

### Quantitative real-time PCR

Quantitative real-time PCR (qPCR) was performed using the SYBR Green-based PCR Kit with the StepOnePlus™ Real-Time PCR System (Applied Biosystems, Foster City, CA, USA). The following primers were used: TNF-α forward: 5′-CTCCTCACCCACACCATCAG-3’, TNF-α reverse: 5′-ATCCCAAAGTAGACCTGCCC-3’; TGF-β forward: 5′-GAGCTGCGTCTGCTGAG-3’, TGF-β reverse: 5′- CCTCAATTTCCCCTCCACGG-3’; CX3CL1 forward: 5′-CCACCTTCTGCCATCTGACT-3’, CX3CL1 reverse: 5′-TCTCCAAGATGATTGCGCGT-3’; ACTB forward: 5′-ACTCTTCCAGCCTTCCTTC-3’, ACTB reverse: 5′- ATCTCCTTCTGCATCCTGTC-3’.

PCR reaction conditions were as follows: 95 °C for 300 s, followed by 40 cycles at 95 °C for 15 s; 60 °C for 40 s and 95 °C for 15 s; 60 °C for 1 min; and 95 °C for 15 s. All experiments were performed in triplicate. Using β-actin (ACTB) as an internal control, the delta-delta Ct method was applied to calculate the relative expression of real-time PCR products.^27^ Threshold cycle (Ct) values were recorded. Relative expression levels of target genes were presented on a log2 scale, and target mRNA levels were expressed as n-fold changes relative to the calibrator. Relative mRNA levels were calculated as 2^Ct^.^28^

### Sample size calculation

Power analysis was performed using PS version 3.0. The smallest difference considered significant between groups in terms of mean change was set at Δ=0.50, α=0.05, and a power of 1-β=0.80. A correlation of ρ=0.20 between replicates was assumed. Based on these parameters, a total sample size of at least 45 participants was determined, comprising 17 participants in Group I, 18 in Group A, and 10 in the Control.

### Statistical analysis

Statistical analyses were performed using SPSS 20. The Kolmogorov–Smirnov test was applied, and all variables were found to be normally distributed; therefore, parametric tests were used. ANOVA was performed to assess differences among the three independent groups, followed by Tukey’s HSD post-hoc test. Gender distribution was determined using the chi-square test. A significance level of α=0.05 was applied for all tests. Data are presented as mean ± standard error of the mean (SEM). Correlation between independent groups was determined using Pearson’s correlation analysis. Additionally, regression analyses were performed to assess the potential effects of age, gender, and clinical indices on biomarker levels.

## Results

### Demographic findings

The gender distribution and mean age of the participants were as follows: Group I, 11 females and six males, mean age 26.52±8.17; Group A, 12 females and six males, mean age 56.16±11.9; and the Control group, nine females and one male, mean age 28.40±8.68. Group A had a significantly higher mean age compared to the other groups (p<0.05). Overall, 71.1% of participants (n=32) were female, with no significant differences in gender distribution among groups (p>0.05) (Table 1).

**Table 1:** Demographic parameters.

	Group I (n=17)	Group A (n=18)	control (n=10)	p-value	I - C	A - C	I – A
Age(y)							
Mean ±standard deviation	26.52± 8.17	56.16±11.9	28.40± 8.68	0.000*^(a)^	0.885^(b)^	0.000*^(b)^	0.000*^(b)^
Sex, N (%)							
Female	11 (64.7)	12 (66.7)	9 (90)	0.325^(x)^			
Male	6 (35.3)	6 (33.3)	1 (10)				

*Abbreviations: Inflammatory(I), inflammatory gingival overgrowth; Amlodipine(A), amlodipine-induced gingival overgrowth; Healthy control group, n: number of samples, Statistically significance value *=p<0.05, a=ANOVA statistical difference between three groups, b=Statistical difference between Tukey HSD two groups, x=Chi square test

### Clinical findings and GCF volume

Full-mouth and sample-site clinical parameters are presented in Table 2. Statistically significant differences were observed in full-mouth clinical periodontal parameters between the test groups and the control group. The mean values of PI, GI, PD, BOP, and GOI for the full mouth were significantly higher in Groups I and A compared to the control group (p˃0.05). Furthermore, the mean values of GI, PD, BOP, GOI, and GCF volume at the sample sites were significantly higher in Groups I and A compared to control group (p˃0.05). Conversely, the mean PI at the sample sites was significantly higher in Group I than in Groups A and the control.

**Table 2:** Clinical periodontal parameters and GCF volume in the full mouth and sampling site.

(a)				I-A-C	I-C	A-C	I-A
	**Group I (n=17 )**	**Group A (n=18)**	**control (n=10)**	**p-value**	**p-value**	**p-value**	**p-value**
PI	1.54±0.48	1.30±0.43	0.67±0 .44	0.000*^(a)^	0.000*^(b)^	0.003*^(b)^	0.257^(b)^
GI	1.21±0.42	1.06±0.50	0.51±0.35	0.001*^(a)^	0.001*^(b)^	0.008*^(b)^	0.620^(b)^
PD (mm)	2.67±0 .52	3.06±0 .62	2.12±0.39	0.000*^(a)^	0.044*^(b)^	0.000*^(b)^	0.098^(b)^
BOP (%)	61.32±20.94	54.11±26.83	25.01±19.74	0.001*^(a)^	0.001*^(b)^	0.008*^(b)^	0.663^(b)^
GOI	1.65±.27	1.49±.34	0.00±0 .00	0.000*^(a)^	0.663^(b)^	0.000*^(b)^	0.217^(b)^
							
**(b)**							
PI	1.91±0.71	1.21±0.53	0.56±0 .46	0.000*^(a)^	0.000*^(b)^	0.022*^(b)^	0.003*^(b)^
GI	1.75±0.58	1.46±0.56	0.82±0.70	0.002*^(a)^	0.001*^(b)^	0.028*^(b)^	0.357^(b)^
PD (mm)	3.25±0.73	3.54±0.91	2.49±0.44	0.004*^(a)^	0.043*^(b)^	0.003*^(b)^	0.507^(b)^
BOP (%)	82.24±25.50	72.11±29.08	41.20±35.38	0.004*^(a)^	0.003*^(b)^	0.028*^(b)^	0.567^(b)^
GOI	2.08±0.40	1.77±0.49	0.00±0.00	0.000*^(a)^	0.000*^(b)^	0.000*^(b)^	0.068^(b)^
GCF Volume (µl)	2.29±1.29	2.53±0.94	1.10±0.46	0.003*^(a)^	0.015*^(b)^	0.003*^(b)^	0.769^(b)^

*Note: Plaque index (PI); Gingival index (GI); Probing depth (PD); Bleeding on probing (BOP); Gingival overgrowth index(GOI). Data are mean ±standard deviation. *=p<0.05, a=ANOVA statistical difference between the three groups, b=statistical difference between the two groups in Tukey HSD.

### CX3CL1, TNF-α, and TGF-β levels in GCF

Total GCF CX3CL1 levels were significantly higher in Groups I and A than the control group (p<0.05), with Group I showing the highest levels (p<0.05). No statistically significant differences were observed in total TNF-α levels among groups (p>0.05). TGF-β levels in Group I were significantly higher than in the control group (p<0.05). There were no significant inter-group differences in CX3CL1 and TGF-β concentrations (p˃0.05), while TNF-α levels were significantly lower in Group A compared to the control (Table 3).

**Table 3:** Sample site GCF fractalkine, TNF-α, TGF-β levels, concentration and total amount values.

				I-A-C	I-A	I-C	A-C
	**Group I (n = 17 )**	**Group A (n = 18 )**	**control (n = 10 )**	**p-value**	**p-value**	**p-value**	**p-value**
CX3CL1 (ng/ml)	0.81±0.35	0.60±0.35	0.83±0.61	0.264^(a)^	0.340^(b)^	0.987^(b)^	0.365^(b)^
CX3CL1 (pg)	1776.35± 1211.25	1495.83± 1065.86	909±876.29	0.000*^(a)^	0.000*^(b)^	0.002*^(b)^	0.007*^(b)^
TNF-α (ng/ml)	22.55±8.29	18.76±4.27	26.91±8.72	0.019*^(a)^	0.262^(b)^	0.279^(b)^	0.015*^(b)^
TNF-α (pg)	50818.35± 32944.89	46432.16± 17995.83	31751.7± 21302.93	0.171^(a)^	0.866^(b)^	0.155^(b)^	0.316^(b)^
TGF-β (ng/ml)	127.38±31.32	103.78±26.23	120.32±48.60	0.125^(a)^	0.113^(b)^	0.862^(b)^	0.441^(b)^
TGF-β (pg)	289426.58± 178833.34	264072.16± 126531.46	144201 ± 110387.64	0.046*^(a)^	0.865^(b)^	0.043*^(b)^	0.105^(b)^

*Note: Data are mean ±standart deviation. *=p<0.05, a=ANOVA statistical difference between three groups, b=Tukey HSD statistical difference between two groups

### Tissue levels of CX3CL1, TNF-α, and TGF-β

Gingival tissue levels of CX3CL1, TNF-α, and TGF-β were presented as fold changes of their relative mRNA expressions at the sampling sites. CX3CL1 and TNF-α levels were significantly higher in Group I compared to Group A (p<0.05), while no significant inter-group difference were observed for TGF-β levels (p˃0.05) (Table 4).

**Table 4:** Levels of tissue fractalkine, TNF-α, and TGF-β.

	Gorup I (n = 17 )	Group A (n = 18 )	control (n = 10 )	I-A-C	I-A	I-C	A-C
				**p-value**	**p-value**	**p-value**	**p-value**
CX3CL1	2.59±1.91	0.54±0.68	1.42±1.30	0.000*^(a)^	0.000*^(b)^	0.103^(b)^	0.258^(b)^
TNF-α	1.85±1.42	0.47±0.53	1.33±1.15	0.002*^(a)^	0.002*^(b)^	0.466^(b)^	0.120^(b)^
TGF-β	1.65±1.13	0.76±0.98	1.60±1.60	0.071*^(a)^	0.086^(b)^	0.993^(b)^	0.196^(b)^

*Note: Data are mean ±standard deviation. *=p<0.05, a=ANOVA statistical difference between three groups, b=Tukey HSD statistical difference between two groups.

### Correlation analysis of clinical and biochemical parameters

In Group I, significant correlations were observed between total GCF CX3CL1, TNF-α, and TGF-β and GCF volume, (r=0.865, r=0.845, and r=0.651, respectively). Additionally, CX3CL1 levels were significantly correlated with TNF-α and TGF-β concentrations (r=0.745 and r=0.647, respectively) (p<0.05). Furthermore, a positive correlation was noted between tissue CX3CL1 and TNF-α and TGF-β levels (r=0.689 and r=0.903, respectively) (p<0.05).

In Group A, a positive correlation was found between tissue CX3CL1 and TNF-α (r=0.762) (p<0.05). Moreover, there was a positive correlation between total GCF CX3CL1 levels and GI (r=0.644), BOP (r=0.622), and GCF volume (r=0.720) (p<0.05).

In the control group, a positive correlation was observed between total GCF CX3CL1 and total TNF-α, total TGF-β, TNF-α concentration, and TGF-β concentration (r=0.782, r=0.825, r=0.743, and r=0.796, respectively) (p<0.05). Furthermore, significant positive correlations were found between tissue CX3CL1 and TNF-α levels, TGF-β levels, and sample site PI values (r=0.850, r=0.833, and r=0.643, respectively) (p<0.05) (Table 5).

**Table 5:** Correlation analysis of Group I, Group A, and Control GCF and tissue samples.

Inflammatory	GCF CX3CL1 (t) - GCF TNF-α (t)	(r=0.865) **
	GCF CX3CL1 (t) - GCF TGF-β (t)	(r=0 .845)**
	GCF CX3CL1 (t) - GCF (v)	(r=0.651)**
	GCF CX3CL1 (c) - GCF TNF - α (c)	(r=0.745) **
	GCF CX3CL1 (c) - GCF TGF- β (c)	(r=0.647)**
	TISSUE CX3CL1 - TISSUE TNF-α	(r=0.689)**
	TISSUE CX3CL1 - TISSUE TGF-β	(r=0.903)**
**Amlodipine**	GCF CX3CL1 (t)- Gİ	(r=0.644)**
	GCF CX3CL1 (t)- BOP	(r=0.622)**
	GCF CX3CL1 (t)- GCF (v)	(r=0.720)**
	TISSUE CX3CL1 - TISSUE TNF-α	(r=0.762)**
**Control**	GCF CX3CL1 (t) - GCF TNF-α (c)	(r=0.743)**
	GCF CX3CL1 (t) - GCF TGF-β (c)	(r=0.796 )**
	GCF CX3CL1 (t) - GCF TNF-α (t)	(r=0.782)**
	GCF CX3CL1 (t) - GCF TGF-β (t)	(r=0.825)**
	TISSUE CX3CL1 - Pİ	(r=0.643)*
	TISSUE CX3CL1 - TISSUE TNF-α	(r=0.850)**
	TISSUE CX3CL1 - TISSUE TGF-β	(r=0.833)**

*Statistically significance value is *: p< 0.05, **: p <0.01, r: Pearson correlation coefficients. Abbreviations: (t) total (c) concentration (v) volume

### Regression analysis

Regression analysis was conducted to investigate whether differences in demographic findings affected dependent and independent variables. The effects of independent variables (age, gender, PI, GI, PD, BOP, GOI, and GCF volume) on markers were analyzed across all groups. In the inflammatory GO group, an increase in GCF volume significantly increased GCF TNF-α, GCF TGF-β, and GCF CX3CL1 levels (p<0.05). GOI was identified as the variable with the strongest effect on tissue TNF-α levels. Three variables—age (p=0.008), GOI (p=0.036), and GCF volume (p=0.046)—had a statistically significant effect on tissue TGF-β levels. Age was also positively associated with tissue CX3CL1 levels (p=0.020), indicating that increasing age increases tissue CX3CL1 expression. GOI showed borderline statistical significance and a negative effect on tissue CX3CL1 levels (p=0.051). In the amlodipine group, PD (p=0.034), GOI (p=0.030), and GCF volume (p=0.006) had a statistically significant effect on GCF TNF-α levels (p<0.05). Among independent variables, GCF volume also significantly affected GCF TGF-β (p=0.006) and GCF CX3CL1 (p=0,045) levels. No independent variable had a significant effect on tissue TNF-α, tissue TGF-β, or tissue CX3CL1 levels (p>0.05). In the control group, GCF volume (p=0.014) was the only variable with a significant effect on GCF TNF-α and GCF TGF-β. Detailed results are shown in Table 6.

**Table 6:** Regression model results.

	Depended Variable	Independed Variables	Unstandardized Coefficients		Standardized Coefficients		
			B	SE	Beta	t	p
		Constant					
Inflammatory	TISSUE TGF-β	Age	0,133	0,038	0,955	3,47	**0,008**
	TISSUE CX3CL1	Age	0,211	0,073	0,897	2,882	**0,02**
	TISSUE TNF-α	GOI	-2,397	1,042	-0,679	-2,301	**0,05**
	TISSUE TGF-β	GOI	-1,744	0,693	-0,621	-2,515	**0,036**
	TISSUE CX3CL1	GOI	-3,049	1,327	-0,642	-2,297	**0,051**
	GCF TNF-α (t)	GCF (v)	20887,68	6271,67	0,82	3,33	**0,01**
	GCF TGF -β (t)	GCF (v)	124473,45	25877,74	0,901	4,81	**0,001**
	GCF CX3CL1 (t)	GCF (v)	553,25	223,53	0,592	2,475	**0,038**
	TISSUE TGF-β	GCF (v)	-0,439	0,186	-0,501	-2,357	**0,046**
Amlodipine	GCF TNF-α (t)	PD	-9763,3	3916,31	-0,498	-2,493	**0,034**
		BOP					
	GCF TNF-α (t)	GOI	17081,57	6611,34	0,467	2,584	**0,03**
	GCF TNF-α (t )	GCF (v)	13100,54	3640,55	0,687	3,599	**0,006**
	GCF -TGβ (t)	GCF (v)	106984,74	30228,03	0,798	3,539	**0,006**
	GCF CX3CL1 (t)	GCF (v)	871,23	373,79	0,756	2,331	**0,045**
Control	GCF TNF-α(t	GCF(v)	51168,75	9953,4	1,121	5,141	**0,014**
	GCF -TGβ (t)	GCF(v)	250596,62	67480,4	1,059	3,714	**0,034**

*SE, standart error; B, beta; Statistically significance value is p< 0.05.

## Discussion

This study is the first investigation of CX3CL1 in both GCF and gingival tissue in patients with GO. We evaluated CX3CL1, TNF-α, and TGF-β levels in GCF and tissue samples obtained from biofilm-induced GO, amlodipine-induced GO, and control groups. CX3CL1, TNF-α, and TGF-β levels in GCF were assessed both as total amounts and concentrations. A review of the literature shows that, in GCF studies, data are commonly presented as total amounts, concentrations, or a combination of both.^29^ Because concentration is based on unit volume and is heavily affected by volumetric variations, it introduces additional challenges in presenting GCF data. Concentration is more commonly used for biological fluids with a constant volume, such as serum.^30^ Analyses of GCF are performed on the entire fluid, which has the characteristics of an exudate. For these reasons, it may be useful to report GCF content both as total amounts and concentrations.^25^ In studies that use both data presentation models, it has been observed that the two approaches do not always yield consistent results.^29^ A similar pattern was observed in our study. While no significant differences were observed in CX3CL1 GCF concentrations among groups, significant differences were observed in CX3CL1 GCF total levels. Conversely, although no difference was observed in total TNF-α levels between A-C groups, a significant difference was observed in the concentration level. For TGF-β, no differences were found in concentration levels between I-C groups; however, significant differences in total levels were observed. Similarly, in the amlodipine group, strong correlations were observed between total GCF CX3CL1 levels and GI, BOP, and GCF volume. These findings highlight the importance of using both GCF concentration and total levels. The mean GCF volume in the sample area was significantly higher in the biofilm and amlodipine groups compared to the control group, consistent with reports describing that GCF volume increases with the degree of inflammation.^31^

Studies have reported that biofilm-induced inflammation contributes to the pathogenesis of GO and that the severity of GO increases with the presence of plaque.^32,33^ Many studies have concluded that dental prophylaxis and effective oral hygiene can prevent the onset of GO or reduce its severity.^2,34^ Güncü, et al.^35^ (2007) observed no statistically significant differences in PI, GI, or gingival bleeding time index (GBTI) between patients with and without GO using phenytoin and nifedipine. Miranda, et al.^36^(2001) compared patients using nifedipine with non-users in terms of GI, PI, PD, and GO. They found that the incidence of GO was significantly higher in nifedipine-treated patients compared to the control group, and that PI and GI values also increased. Among potential risk factors, only GI and GO were significantly associated, suggesting that gingivitis may act as a predisposing factor. In our study, clinical parameters—including PI, GI, PD, BOP, and GOI—were higher in GO groups than in the control group, and sample site PI was higher in Group I than in Groups A and C. These findings support the notion that plaque accumulation can trigger gingival enlargement.

Recently, inflammation biomarkers have been utilized as molecular tools for diagnosing periodontal disease, with CX3CL1 and its receptor CX3CR1 considered potentially significant markers.^37^ CX3CL1 and CX3CR1 are involved in both acute and chronic inflammatory processes, amplifying inflammation and exacerbating disease outcomes.^5^ To date, few studies have focused on the role of the CX3CL1–CX3CR1 axis in periodontitis. Balci, et al.^17^ (2021) reported significantly elevated levels of CX3CL1 and CX3CR1 in the GCF of individuals with periodontitis compared to healthy controls, with positive correlations between these inflammatory markers and clinical parameters. Yilmaz, et al.^18^ (2021) observed increased CX3CL1 levels in the saliva of rheumatoid arthritis (RA) patients with periodontitis, as well as elevated serum CX3CL1 levels in patients with both RA and periodontitis compared to other groups. These studies suggest that the CX3CL1–CX3CR1 axis is likely involved in regulating inflammatory processes in the periodontium, especially in recruiting leukocytes to damaged sites.

Garcia, et al.^7^ (2000) showed that membrane-bound CX3CL1 is induced by various pro-inflammatory stimuli, such as IL-1β and TNF-α, in LPS-activated rat aortic endothelial cells, and highlighted its interaction with NF-κB in response to these stimuli. In an *in vitro* study, Wang, et al.^38^ (2014) reported an association between CX3CL1/CX3CR1 and the progression of periapical lesions in rats, suggesting their involvement in tissue damage, including bone resorption, during periapical inflammation. Additionally, Hosokawa, et al.^15^ (2005) showed that leukocytes in periodontal disease tissues express CX3CR1, while the ligand chemokine CX3CL1 is strongly expressed in endothelial cells within these tissues. Upregulation of CX3CL1 expression has been observed in response to pathogen-associated molecular patterns (PAMPs), including *P.gingivalis* LPS, indicating that the CX3CL1–CX3CR1 system may contribute to leukocyte infiltration in periodontal tissues and the progression of periodontal disease.^15^ Consistent with these findings, our study demonstrated increased CX3CL1 and TNF-α expression in gingival tissue samples exhibiting inflammatory overgrowth. Moreover, total GCF CX3CL1 levels were significantly higher in the biofilm and amlodipine groups compared to the control group.

In the amlodipine-induced GO group, a strong positive correlation was observed between total GCF CX3CL1 levels and GI, BOP, and GCF volume. GI and BOP are clinical parameters that reflect the degree of inflammation. This inflammatory response may result from the direct toxic effects of concentrated drugs and/or the presence of bacterial plaque in GCF, leading to increased levels of several cytokine.^39^ These findings indicate that elevated CX3CL1 levels in GCF may indicate localized tissue inflammation.

TNF-α is as a potent inducer of matrix metalloproteinases (MMPs), which contribute in collagen metabolism within the ECM.^40^ While no significant differences were observed in total GCF TNF-α levels among the study groups, GCF TNF-α concentration was significantly lower in the amlodipine group than controls. This may be associated with the increased fibrotic nature of gingival tissue in the amlodipine group. Furthermore, TNF-α detected in GCF samples of healthy individuals may originate from neutrophils, macrophages, and mononuclear cells naturally present in gingival tissues.^41^ Subclinical gingivitis may also be present in clinically healthy patients, contributing to detectable TNF-α levels associated with inflammation.

TGF-β, widely recognized as a regulator of the ECM, is associated with fibrosis in various diseases.^42^ TGF-β1 levels in GCF of patients with drug-induced GO have been suggested as a possible indicator of GO.^43^ Research in animal models of fibrosis indicates that the CX3CL1–CX3CR1 signaling pathway directly influences collagen production.^13,44^ Shimizu, et al.^44^ (2011) demonstrated that the CX3CL1–CX3CR1 pathway contributes to renal fibrosis in a mouse model of hypertension, likely by promoting macrophage infiltration and increasing TGF-β1 and type I collagen expression. Research in mice has further demonstrated that the CX3CL1–CX3CR1 axis is key in the accumulation of macrophages and fibroblasts at wound sites.^45^ In a murine model of bleomycin-induced pulmonary fibrosis, the CX3CL1–CX3CR1 axis has been reported to exert a pro-fibrotic role.^46^ In a series of *in vitro* experiments on synovium-associated fibroblasts in osteoarthritis patients, Klosowska et al. demonstrated that CX3CL1 has a chemotactic effect on fibroblasts, inducing their migration along the CX3CL1 concentration gradient.^47^ Similarly, Buskermolen, et al.^8^ (2017) discovered that CX3CL1–CX3CR1 stimulated migration and IL-6 secretion of gingiva fibroblasts. Virulence factors from periodontopathogenic bacteria also promote CX3CL1 production by gingival keratinocytes and fibroblasts.^37^ In our study, total GCF TGF-β levels were significantly higher in the inflammatory GO group compared to controls (p<0.05). We identified the highest correlation between tissue CX3CL1 and TGF-β levels (r=0.903) in the inflammatory GO group. These findings suggest a potential role for fractalkine in the development of tissue fibrosis associated with GO, alongside its inflammatory mechanisms. However, additional research is required to elucidate these intricate mechanisms.

We determined that total GCF CX3CL1 levels, as well as tissue CX3CL1 and TNF-α levels, were higher in the inflammatory GO group than in the amlodipine-induced GO group. These differences may be related to the higher mean plaque index in the sample area of the inflammatory GO group. However, a recent cross-sectional study examining the chemokine and subgingival microbial profiles of periodontal health reported low CX3CL1 expression in the GCF of healthy individuals. This suggests that the inflammatory response in a healthy host is dynamic and variable, and may not be directly linked to the oral microbial composition.^48^

The interaction between CX3CL1 and amlodipine requires further investigation to identify possible treatments for GO. As a limitation of our study, only ligands of relevant chemokines were evaluated in GCF and tissue samples. Future studies should evaluate the receptor, its binding to ligands, and the second- or third-messenger pathways activated, to elucidate the detailed action/activity of CX3CL1. Furthermore, *in vitro* or *in vivo* studies with larger sample sizes, evaluating the CX3CL1–CX3CR1 axis in both health and disease conditions, are needed to better reveal its role in GO pathogenesis. Finally, no previous studies have reported CX3CL1 levels in GCF and gingival tissue in patients with GO; thus, our data could not be compared with similar studies.

## Conclusion

CX3CL1 and TNF-α levels were higher in gingival tissue samples of patients with biofilm-induced GO compared those with amlodipine-induced GO. GO negatively impacts patients’ quality of life and may indirectly affect systemic health. Considering the unexplored role of CX3CL1 in GO and its known involvement in fibrosis and inflammatory processes, investigating the interaction between CX3CL1 and its receptor is crucial for identifying potential therapeutic targets.
